# Fine‐scale foraging behavior reveals differences in the functional roles of herbivorous reef fishes

**DOI:** 10.1002/ece3.7398

**Published:** 2021-03-18

**Authors:** Robert F. Semmler, Simon J. Brandl, Sally A. Keith, David R. Bellwood

**Affiliations:** ^1^ Lancaster Environment Centre Lancaster University Lancaster UK; ^2^ Department of Marine Science Marine Science Institute University of Texas Austin Port Aransas TX USA; ^3^ ARC Centre of Excellence for Coral Reef Studies James Cook University Townsville QLD Australia; ^4^ College of Science and Engineering James Cook University Townsville QLD Australia

**Keywords:** complementarity, coral reefs, foraging behavior, functional traits, movement

## Abstract

Efforts to understand and protect ecosystem functioning have put considerable emphasis on classifying species according to the functions they perform. However, coarse classifications based on diet or feeding mode often oversimplify species' contributions to ecological processes. Behavioral variation among superficially similar species is easily missed but could indicate important differences in competitive interactions and the spatial scale at which species deliver their functions. To test the extent to which behavior can vary within existing functional classifications, we investigate the diversity of foraging movements in three herbivorous coral reef fishes across two functional groups. We find significant variation in foraging movements and spatial scales of operation between species, both within and across existing functional groups. Specifically, we show that movements and space use range from low frequency foraging bouts separated by short distances and tight turns across a small area, to high frequency, far‐ranging forays separated by wide sweeping turns. Overall, we add to the burgeoning evidence that nuanced behavioral differences can underpin considerable complementarity within existing functional classifications, and that species assemblages may be considerably less redundant than previously thought.

## Graphical abstract

Broad functional groupings may mask important behavioral nuances that could affect competitive interactions and the spatial scale at which species deliver their functions. We find considerable variation in foraging patterns and spatial scales of operation both within and across traditional functional groups (grazers and scrapers). This highlights substantial complementarity within the grazer group, indicating that together these species may contribute more strongly to algal grazing than any could alone.

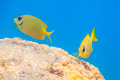

## 1 INTRODUCTION

Understanding how, why, and when different species contribute to essential ecosystem functions has gained increased focus in recent years, with the aim to both advance fundamental knowledge and improve management (Bellwood et al., 2019; Díaz & Cabido, 2001; Folke et al., 2004; Tilman et al., 1997). For both fundamental and applied research, it is not only important to identify species that are key to the maintenance of essential functions, but also to establish the extent to which species are functionally similar (underpinning redundancy) or different (underpinning complementarity) (Blüthgen & Klein, 2011; Brandl et al., 2019; Burkepile & Hay, 2011; Frost et al., 1995; Lawton & Brown, 1993; Nyström, 2006). Complementarity essentially describes niche partitioning in an Eltonian, functional context (Bellwood et al., 2019; Brandl et al., 2019). Substantial complementarity has been documented within superficially homogeneous groups of flying insect pollinators (Blüthgen & Klein, 2011), grazing subtidal urchins (Brandt et al., 2012), savannah ungulates (Pringle et al., 2014), and small desert herbivores (Thibault et al., 2010). However, for practical purposes a delicate balance is necessary between the benefits of tractability and the risks of oversimplification. On the one hand, it is necessary to ensure tractability or utility of functional groups, which requires collapsing diverse species into groups of ecologically similar entities, for example, trophic groups or guilds. On the other hand, groupings may oversimplify ecological dynamics, masking important differences between species within the same functional category and their contributions to ecological processes (Körner, 1994).

Ecosystems with high inherent species richness, such as coral reefs and tropical rainforests are characterized by a complex mosaic of biological interactions, and a wide variety of available of microhabitats (Gentry, 1982; Graham & Nash, 2013; Reaka‐Kudla, 1997). This complexity has spurred the development of functional group classifications, on coral reefs in particular (Bellwood et al., 2004; Darling et al., 2012; Nyström, 2006). Nevertheless, species within these groups may differ in a number of ways that could impact the delivery of their functions. Thus, to ensure that functions are maintained as species assemblages change, we need to know the extent to which species within the same broad functional entity differ from one another. It is doubtful that there is “true redundancy” within functional groups; rather there will be some degree of complementarity, dependent on the scale at which behavior is assessed (Brandl & Bellwood, 2014). Within functional entities, complementarity of functional delivery can be a result of fine‐scale partitioning of resources, which can be based on species‐specific differences in targeted resources, or temporal and spatial patterns in their exploitation (Fründ et al., 2013; Wellborn & Cothran, 2007). Species foraging patterns are likely to reflect all of these elements, thus providing a window into the extent of functional complementarity among species.

Foraging movements are determined by economic decisions to optimize the food resource gained per unit of energy expended (MacArthur & Pianka, 1966). Thus, while not the only factors affecting movement, foraging movements depend both on dietary preferences and the abundance and patchiness of the food resources targeted (Stephens & Krebs, 1986). For example, to account for long travel times and their associated costs, patchy food resources require long patch residence times (Charnov, 1976). Additionally, low‐quality patches will be depleted quickly below an energy gain per unit effort that maintains optimum foraging (McNair, 1982). As a result, species that focus their diets on patchy or lower quality food items may have shorter patch residence times and greater exploration times (Stephens & Krebs, 1986). Because foraging movement decisions are made based on the density and location of food resources, even among closely related species, these types of small differences in dietary preference can favor different foraging strategies (Pyke, 1984). In addition to these factors, patch use may also be impacted by the threat of predator (Brown et al., 1992; Catano et al., 2015) or competitor species (Mitchell et al., 1990).

Variations in foraging strategy are the result of adaptive changes that facilitate coexistence among species competing for space and resources (Chesson, 2000; Tilman, 1982). However, different foraging strategies are also likely to affect the spatial extent over which species perform their role (Nash et al., 2013, 2016). Efforts to identify a forager's spatial scale of operation through home‐range assessments are useful but feeding activity can be heterogenous and concentrated within certain areas of the animal's range (Streit et al., 2019; Welsh & Bellwood, 2012). As a result, assessments of animals' foraging movements can benefit from various types of behavioral observations across multiple spatial and temporal scales.

Coral reef fishes can overlap heavily in their broad use of habitats and in their contributions to ecosystem functions (Mouillot et al., 2014). Conservation actions have been adopted on the basis of these strategies to manage coral reef ecosystems with a particular focus on the role of herbivorous fishes (Adam et al., 2015a, 2015b; Chung et al., 2019; Green & Bellwood, 2009). Herbivory by coral reef fishes was originally divided into four broad functional categories based on foraging strategies: grazers, browsers, scrapers, and bioeroders (Bellwood et al., 2004; Green & Bellwood, 2009; Nyström, 2006). These categories cover a suite of functions that facilitate reef resilience to disturbance, and can prevent them from shifting to less desirable, alternate states dominated by algae (Hughes et al., 2007). However, species within these groups are far from homogenous in their niches (Bellwood et al., 2019; Brandl et al., 2019). For example, browser species can differ strongly in their preference for algal food resources (Puk et al., 2016; Rasher et al., 2013; Streit et al., 2015), while grazers separate into species targeting the tips of algae (e.g., croppers) and species targeting particulate matter within algal turfs (e.g., Brandl & Bellwood, 2016; Tebbett et al., 2017). Similarly, scraping and bioeroding parrotfishes differ substantially in their ingestion and postingestion treatment of resources (Adam et al., 2018; Clements et al., 2016; Nicholson & Clements, 2020), leading to various refinements of the initial categories over the years (Brandl & Bellwood, 2016; Siqueira et al., 2019). However, these classifications still focus primarily on diet and resources acquisition method. Few consider spatial dimensions of resource use. While reef herbivores are known to vary in their specific microhabitat use (e.g., horizontal, vertical, underside) (Adam et al., 2018; Brandl & Bellwood, 2014; Fox & Bellwood, 2013; Puk et al., 2020), fine‐scale foraging movements and spatial resource partitioning in coral reef fishes remains poorly understood (Streit et al., 2019). Yet it is at this scale that resource partitioning and complementarity may be most strongly expressed, with significant effects for reef functioning (Ruttenberg et al., 2019).

We investigate the degree to which differences in foraging behavior can transcend boundaries set by traditional functional group classifications. Specifically, we assess the fine‐scale foraging movements of three coral reef herbivores: two grazer/cropper species (*Siganus corallinus* and *Siganus vulpinus*) and one scraper (*Scarus schlegeli*). We ask: How does foraging behavior and space use vary between species? Specifically, (a) which traits (speed, turning angle etc.) define the differences between their foraging paths? (b) Do short‐term hourly movement patterns (in situ behavioral observations) reflect longer‐term daily patterns of space use (assessed via active acoustic telemetry)? (c) Are there substantial differences in the scale of operation among species, and does this affect the spatial extent over which these species perform their functional role?

## 2 METHODS

**FIGURE 1:**
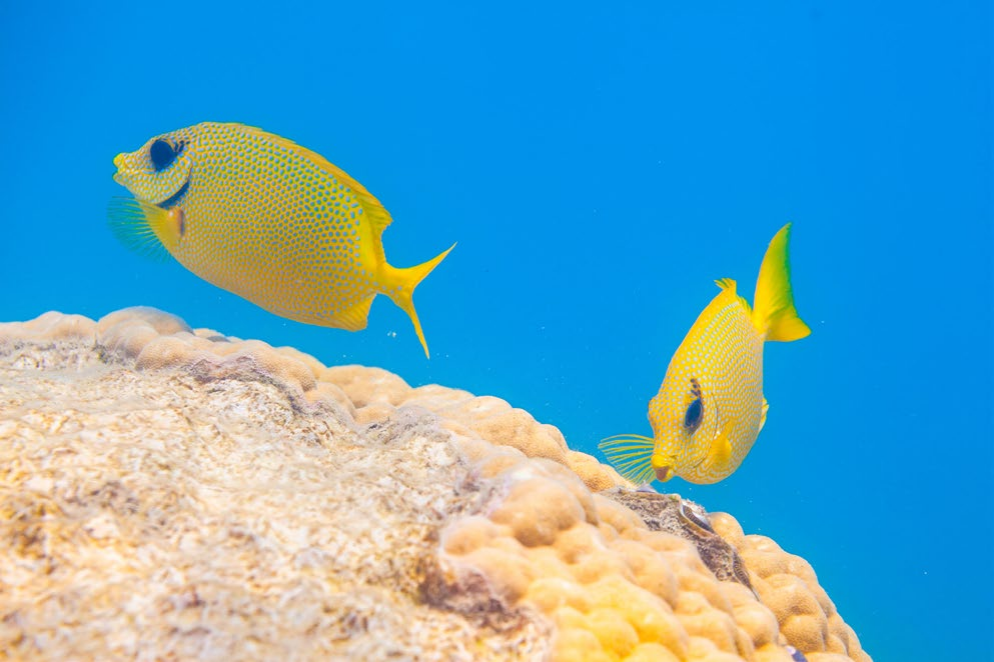
Photo of two *Siganus corallinus* individuals (credit: Victor Huertas)

Field sites were located on reefs at Lizard Island, a granitic mid‐shelf island on the Great Barrier Reef. We studied three species: two rabbitfishes *Siganus vulpinus* and *Siganus corallinus* (Figure 1), and one parrotfish *Scarus schlegeli*. The two rabbitfishes are categorized as cropping herbivores that take discrete bites from small algae or cyanobacteria (i.e., grazers) (Brandl & Bellwood, 2016; Hoey et al., 2013) and occur almost exclusively in stable pairs (Brandl & Bellwood, 2013; Brandl & Bellwood, 2015). By contrast, *Scarus schlegeli* lives in small groups and is a scraping herbivore that ingests the entire epilithic algal matrix (i.e., scraper) (Clements et al., 2016). While the vast majority of grazing herbivores on reefs have limited home ranges and exhibit strong site fidelity at the reef scale, there is considerable variation in the movements among both rabbitfishes (Brandl & Bellwood, 2013; Fox & Bellwood, 2011) and parrotfishes (Welsh & Bellwood, 2011, 2012). The three species in the present study were selected to permit a comparison between two species commonly considered to be functionally equivalent (the two cropping rabbitfishes), while anchoring these observations within the broader classification of grazing herbivores by including a functionally different species (the scraping parrotfish). Foraging path observations were performed on Big Vicki's Reef (5 hectares) from February 7th to February 11th 2014, while the acoustic tracking was performed on Watson's Reef (2 hectares) from April 25th to May 4th 2012. Both reefs are on the leeward side of the island and represent typical backreef sites with low wave energy and depths between 2 and 5 m. The two reefs are separated by a distance of approximately 2 km and represent broadly similar lagoonal habitats dominated by corals and turf algae. We chose to perform the two parts of the study on different reefs for several reasons: (a) since acoustic tracking involves the capture and manipulation of fishes, which may modify the individual's reactions to observers in the water, we considered it safer to avoid the reef that fishes were tagged on; (b) Big Vicki's reef offered a more expansive and slightly deeper reef environment, thus allowing for higher replication without the risk of re‐sampling the same individuals, while ensuring a minimal observer effect from the snorkeler in the water.

### 2.1 Focal foraging path observations

We quantified the fishes' foraging movements in situ. A single snorkeler (SJB), equipped with a handheld global positioning system (GPS) unit in a waterproof case, which was set to automatically record its position every 5 s, performed the observations. We opportunistically located an adult of one of the three target species and followed the fish for 30–45 min. We followed the fish as closely as possible (snorkeling offering one of the least disturbing methods of observation; Welsh & Bellwood, 2011), recording different behaviors (i.e., swimming and feeding behavior). For each behavior, the observer recorded the exact time of the event (hh:mm:ss) using a digital wristwatch that was precisely synchronized with the GPS unit. All focal observations occurred between 08:00 and 17:00, a time window during which most herbivorous fish species are actively foraging. We considered a foraging bout to be finished once the fish stopped biting the substratum and assumed a horizontal position characteristic of swimming activity (Nash et al., 2012). During all observations, we ensured positioning directly above the focal individual (which restricted our observations to areas with depths >2 m to ensure fishes were undisturbed by the observer). After 30–45 min (or when the focal individual showed signs of behavioral modification due to being followed by the snorkeler or contact was lost due to depth or visibility), the observer abandoned the focal individual in search of an individual of one of the other two species. Once individuals in all three species were followed, the observer took a haphazard turn, swam for at least 100 m, and searched for another individual in any of the three target species. To avoid duplication, we spread efforts across different sections of the reef and took notes on size and color patterns of the observed fish.

### 2.2 Acoustic telemetry

To obtain a more detailed assessment of space use in the two rabbitfish species, we used active acoustic telemetry on five adult individuals of *Siganus corallinus* (in three pairs; SC1 and SC2, SC4, SC5, and SC6) and three adult individuals of *Si*. *vulpinus* (in two pairs; SV2, SV3 and SV4). An additional individual was tagged in each species but disappeared shortly after release, probably due to predation (Khan et al., 2016). While the behavior of paired individuals will not be wholly independent from their partner, separation of individuals or exclusive treatment of only one partner can result in changes of behavioral patterns. To tag the individuals, we caught pairs using barrier nets on Watson's Reef and transported them immediately to Lizard Island Research Station in large bins full of fresh seawater, ensuring pairs were maintained. At the station, we placed pairs in separate large (300 L) flow‐through seawater aquaria. In the evening of the day of capture, we anesthetized each fish in a saline solution of tricaine methanesulfonate (MS‐222, 0.13 g/L) and surgically implanted an acoustic transmitter (Vemco V9‐6L) into the gut cavity (cf. Brandl & Bellwood, 2013). After closing the incision with sutures and ensuring full recovery from anesthesia, we held fishes in their tanks overnight. We returned the fishes to the exact site of capture the next morning.

Fish were allowed 48 hr to recover, after which we started acoustically tracking each fish from a 3.1 m kayak using a calibrated directional hydrophone (VH110) and an acoustic receiver (VR100, both Vemco) (Brandl & Bellwood, 2013; Fox & Bellwood, 2011). Tracking continued from 30 min before dawn to 30 min after dusk (approx. 06:30–18:00). We maneuvered the kayak to obtain maximum signal strength from the respective tag every 15 min, while the receiver recorded the kayak's GPS position. We tracked each fish for three nonconsecutive days and verified the identity and normal behavior of the tracked individual via a short in situ validation by a snorkeler each day (identifying the tagged fishes through the visible surgical incision; Brandl & Bellwood, 2013).

### 2.3 Data analysis

We performed all data analyses in R (R Core Team, 2019). For the snorkeler‐based observations, we matched timed GPS recordings with recorded times for each feeding event to quantify the path between successive feeding events for each. From these, we calculated six traits to characterize different aspects of foraging behavior or space use: (a) 95% minimum convex polygon (MCP) of space used during the observation, (b) mean swimming speed, (c) mean turning displacement (higher displacement = sharper turns) between successive movement bearings, (d) overall tortuosity of the feeding path, (e) number of feeding events per minute, and (f) average distance between feeding events (interforay distance). We computed MCPs using the package *adehabitatHR* (Calenge, 2006), and distances (using the Haversine method) and bearings between points, using the package *geosphere* (Hijmans, 2016). We calculated overall path tortuosity as the ratio of the straight‐line distance between the start and end locations, and the total distance travelled by the fish (following Fulton & Bellwood, 2002; Secor, 1994). We tested for differences between the three species in each of these traits with Analyses of Variance (ANOVA). To ensure normality and homoscedasticity of variances, it was necessary to log transform the MCP values. After transformation, MCP values for *Si*. *vulpinus* became normally distributed (Shapiro–Wilk: 0.88, *p* = 0.12) and MCP variance among species was homogenous (Bartlett: 5.64, *df* = 2, *p* = 0.06).

Furthermore, we visualized inter‐ and intraspecific variation in these traits with a nonmetric multidimensional scaling (MDS) ordination based on a Bray–Curtis dissimilarity matrix (Gauch, 1973). We ran the ordination on a square root Wisconsin transformed matrix to ensure that differences in scale between trait values did not influence the analysis (Del Moral, 1980). We used a Permutational Analysis of Variance (PERMANOVA) to test for significant differences in the overall foraging strategies of the three species and tested for homogeneous multivariate dispersion between species using PERMDISP. Lastly, we used the SIMPER analysis to determine which traits contributed most to differences in foraging behavior between species. PERMANOVA, PERMDISP, and SIMPER tests were run on the transformed dissimilarity matrix using the package *vegan* (Oksanen et al., 2016).

For the active tracking data, we used the GPS points from each 15‐min intercept (choosing the highest‐strength signal around the 15‐min mark) to compute kernel utilization distributions (KUDs) for each individual, which we used to estimate 95% daily foraging areas and 50% core areas for each individual (Brandl & Bellwood, 2013). We calculated KUDs for each day and the cumulative GPS points across all days. We again used the package *adehabitatHR* (Calenge, 2006). We tested differences in cumulative daily foraging areas and core areas between the two rabbitfishes with two‐sample *t* tests.

Lastly, we also computed overall feeding rates (bites/min) and movement rates (meters/min) for each fish observed on snorkel. Specifically, our rationale was that differences in foraging strategy between species may be underpinned by fine‐scale dietary differences. Differences in feeding efficiency between species may help to highlight this, as diets may provide more or less energy per bite. Feeding rates were calculated based on the total time spent feeding within each observation (with each feeding event estimated as 5 s), multiplied by previously established bite rates during feeding events, for each species (Brandl & Bellwood, 2014). Feeding efficiency was calculated by dividing each individual's feeding rate by its movement rate. As with the six traits above, for these three factors we tested differences between species with ANOVA.

## 3 RESULTS

**FIGURE 2:**
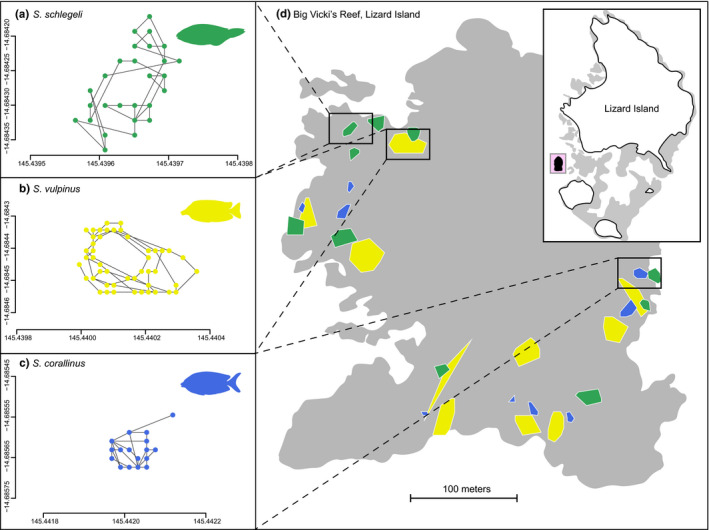
Foraging paths and resulting size and distribution of short‐term feeding areas (direct observation). (a–c) Example foraging paths for all three species. Green = the parrotfish *Sc*. *schlegeli*, yellow and blue = the rabbitfishes, *Si. Vulpinus*, and *Si. corallinus*, respectively. Dots represent foraging locations, while lines represent vectors between foraging events. Path insets not scaled by area, but relative size can be seen in the wider figure. (d) Distribution of feeding areas (MCP) for each species on Big Vicki's Reef with inset showing location of Big Vicki's Reef on Lizard Island, colors as above

**FIGURE 3:**
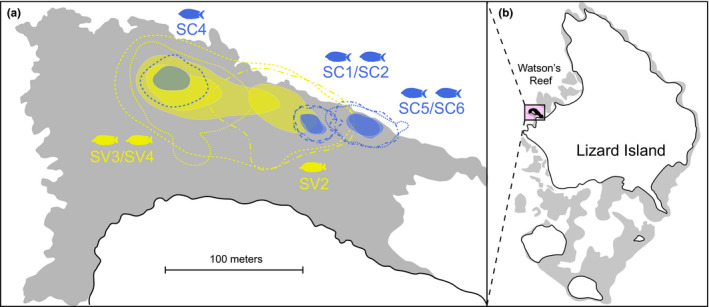
Relative size of daily foraging areas (acoustic telemetry). (a) Spatial distribution of daily foraging areas on Watson's Reef. Dotted and dashed lines mark the 95% Kernel Utilization Distributions (KUDs), while filled, transparent areas mark the 50% core areas. Fish numbers are given for all paired and the two singular individuals. Colors as above. (b) Location of Watson's Reef on Lizard Island

**FIGURE 4:**
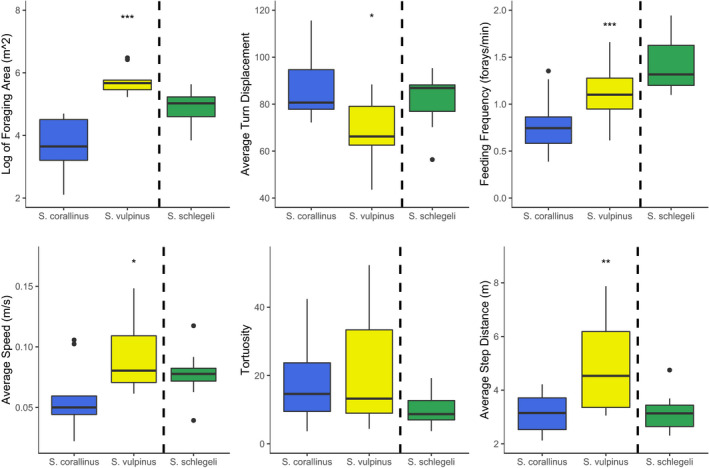
Differences in the six metrics used to evaluate foraging paths of the three species. Asterisks indicate significant differences among species via ANOVA. *Si*. *vulpinus* foraging movements are characterized by: large areas, wide turns, higher speeds, and longer interforay distances; *Si*. *corallinus* foraging movements are characterized by: small areas, sharp turns, low feeding frequency, low speed, and short interforay distances; *Sc*. *schlegeli* occupy intermediate positions but display the highest frequency of foraging. Boxplots represent the median and interquartile range of each foraging trait. Dashed lines separate the two grazing rabbitfishes from the scraping parrotfish

Overall, we followed 29 individual fishes (counts: *Siganus corallinus* = 9 individuals; *Si. vulpinus* = 10 individuals; *Scarus schlegeli* = 10 individuals). Overall observation time totaled 17.4 hr (mean observation times: *Siganus corallinus* = 35.9 min ± 2.21 SE; *Si. vulpinus* = 34.5 min ± 2.54; *Scarus schlegeli* = 37.4 min ± 1.82) during which we recorded 1,190 feeding events. Foraging patterns differed for the three fish species, both within and across functional group boundaries. Variation in short‐term foraging movements (Figure 2) was mirrored by daily space use in the two rabbitfishes, where both 95% daily foraging areas and 50% core areas of *Si*. *vulpinus* were significantly larger than those of *Si*. *corallinus* (*t*(6) = −6.00, *p* < 0.001, and *t*(6) = −6.28, *p* < 0.001, respectively) (Figure 3). Overall, we found significant variation between species for five of the six movement traits we investigated (Figure 4). Specifically, there were significant differences in the log of foraging area covered (*F*_2,26_ = 21.96, *p* > 0.001), mean speed of travel (*F*_2,26_ = 3.98, *p* = 0.031), mean turn angle (*F*_2,26_ = 4.71, *p* = 0.018), feeding frequency (*F*_2,26_ = 9.44, *p* > 0.001), and mean interforay distance (*F*_2,26_ = 7.41, *p* = 0.003). *Si*. *corallinus* had the smallest mean foraging area, while *Si*. *vulpinus* had the largest. We found a similar relationship for mean speed, with *Si*. *vulpinus* travelling at greater speeds than *Si*. *corallinus*. *Si*. *vulpinus* also took wider turns between feeding bouts compared to *Si*. *corallinus* and *Sc. schlegeli* (Figure 4). However, despite difference in turning angles, we found no significant differences for the overall tortuosity of foraging paths. While mean tortuosity did not differ, variance in path tortuosity was substantially larger for the rabbitfishes than for *Sc. schlegeli*. *Sc*. *schlegeli* had more frequent foraging bouts than *Si*. *corallinus*, and *Si*. *vulpinus* had longer interforay distances than either of the other species.

**FIGURE 5:**
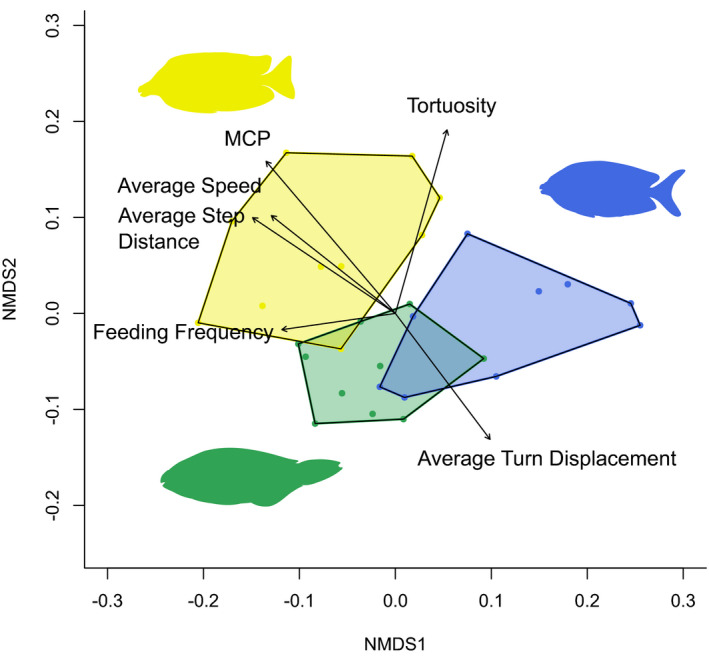
Nonmetric multidimensional scaling ordination depicting differences in foraging paths of three species: the rabbitfishes *Siganus vulpinus* (yellow), *Si*. *corallinus* (blue), and the parrotfish *Scarus schlegeli* (green). Convex hulls represent minimum convex polygons for all individuals of a species. Vectors represent the loadings

As would be expected from the results above, species identity was significant in determining foraging behavior, explaining 42% of variance among individuals (*R* ^2^ = 0.42, *p* < 0.001, Figure 5). All species showed similar levels of intraspecific variability in foraging traits; multivariate dispersions were not significantly different between species (*p* = 0.060). Despite not differing significantly in the univariate analysis, path tortuosity contributed to differences between species within the multivariate analysis. Differences between species were most strongly predicted by the size of their foraging areas, the tortuosity of their foraging paths and the mean turning angle between feeding events, with each of these traits explaining over 20% of the difference between any two species. Mean speed was the least informative trait, explaining <10% of the average difference between any two species. Differences between the parrotfish *Sc*. *schlegeli* and the rabbitfish *Si*. *corallinus*, were mostly driven by a tighter (18%), smaller (19%) and more tortuous feeding path (24%) for the rabbitfish. Similar differences were reflected between the two rabbitfish, with large proportions of variance defined by tighter turns (26%), and a smaller feeding area (29%) for *Si*. *corallinus*, however a less tortuous path (20%) than *Si*. *vulpinus*. Differences between *Sc*. *schlegeli* and *Si*. *vulpinus* were also most strongly determined by a larger (19%) more tortuous feeding path (21%) for the rabbitfish, as well as a faster feeding frequency (21%) for the parrotfish.

**FIGURE 6:**
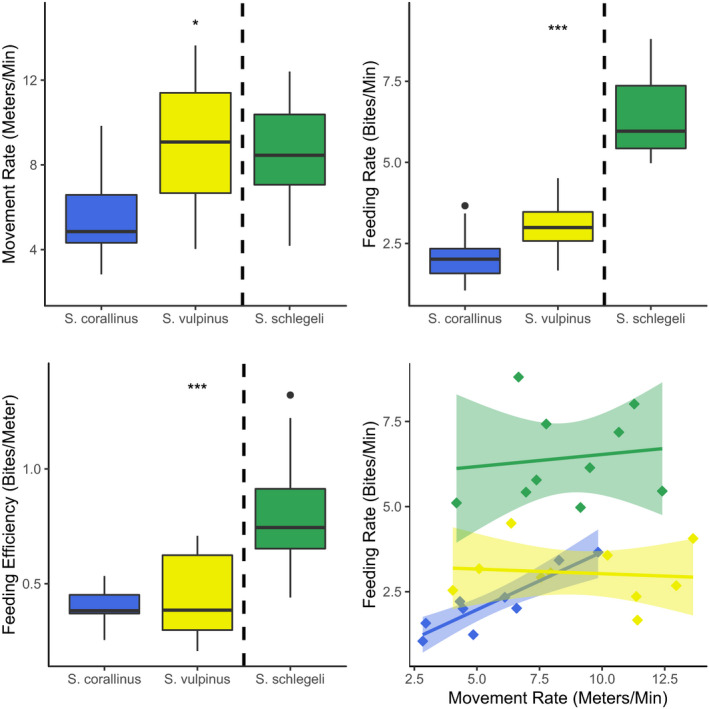
Feeding rate, movement rate, and feeding efficiency of the three species. Asterisks indicate significant differences among species via ANOVA. Boxplots represent the median and interquartile range of each foraging trait. Dashed lines separate the two grazing rabbitfishes from the scraping parrotfish

Lastly, species differed significantly in their feeding rates (*F*_2,26_ = 44.55, *p* > 0.001), movement rates (*F*_2,26_ = 4.33, *p* = 0.024), and their resulting feeding efficiency (*F*_2,26_ = 12.71, *p* > 0.001) (Figure 6). *Si*. *corallinus* had the lowest movement rates, with both *Si*. *vulpinus* and *Sc. schlegeli* moving faster. Based on unique foraging events and bite rates, the parrotfish took many more bites per minute than either rabbitfish species. Due to these differences, the feeding efficiency of the parrotfish was higher than either rabbitfish. While across the three species, a positive relationship between movement and bite rates was visible, only *Si*. *corallinus* showed an intraspecific trend where individuals traveling farther took more bites per unit time.

## 4 DISCUSSION

Categorization of species based on their functional roles is a useful concept in ecology and conservation. However, behavioral differences among species within the same group may result in functional variation that is unaccounted for in broad categories. Our results demonstrate behaviorally mediated diversity in functional roles of herbivorous fishes within and across functional groups, resulting in complementarity in their niches and spatial differences in the delivery of their functional roles. The differences in fine‐scale foraging paths of the grazers, *Si. corallinus* and *Si. vulpinus*, are reflected in their broad‐scale, reef‐scape movements. Both fine‐scale activities and sustained broad‐scale movements are critical components of animals' energy budgets, but they also shape their functional roles within ecosystems, especially in a spatial context.

In our analysis, we found clear differences in foraging behavior between the three fish species, even those within the same functional group and genus, that is, grazing rabbitfish. Feeding frequency was the primary trait that differentiated the two functional groups, both in terms of the number of forays per minute and the number of bites per minute. This difference could be expected as scrapers primarily remove epithelial algal matrix from flat or convex surfaces, which can be more readily located without disrupting movement (Brandl & Bellwood, 2014; Clements et al., 2016). The two grazers, on the other hand, will inspect holes or crevices for patches of algae to crop (Brandl & Bellwood, 2015; Fox & Bellwood, 2013), leading to slower bite rates and less frequent feeding events. As a result of its fast feeding rate and intermediate movement rate, the parrotfish appears to be the most efficient, or least selective, forager, taking the largest number of bites while traveling only short distances between those bites.

As well as the expected behavioral differences between functional groups (i.e., grazers vs. scrapers), there were substantial differences between the two grazers. *Si*. *corallinus* moved slowly, focusing feeding effort within a very small area of the reef, and took sharp turns to stay within this core area. In contrast, *Si*. *vulpinus* ranged widely over a considerably larger feeding territory and travelled substantial distances between forays in a roughly circular, and remarkably predictable pattern. Though sample sizes for acoustic tracking were limited and included nonindependent paired individuals, we have considerable confidence that these differences were reflected in the daily foraging areas of each species as well, with *Si. corallinus* occupying a much smaller foraging area than *Si*. *vulpinus*. Complementary scales of space use among these two species indicate that both species will contribute more strongly to algal grazing than either could alone, which holds important implications for the management of herbivory on coral reefs (Topor et al., 2019).

Some of the differences in the foraging search patterns of the rabbitfish species could be driven by differences in their diets. While both are considered grazers, *Si*. *corallinus* primarily targets small, dense red algae, while *Si. vulpinus* mostly consumes cyanobacteria (Hoey et al., 2013). Furthermore, *Si*. *vulpinus*, with its extremely elongated snout, appears to obtain most of its food from deep crevices and interstitial microhabitats compared to *Si*. *corallinus*, which targets shallower crevices that it can exploit with its more moderate head morphology (Brandl & Bellwood, 2014, 2016). Differences in foraging behavior between the two species may be driven by the spatial organization of these resources on reefs and their patchiness; while small red algae and shallow crevices can be expected to occur frequently throughout the reef matrix, deeper crevices with dense mats of cyanobacterial growth are less common (Brandl, Robbins, & Bellwood, 2015; Harris et al., 2015). These differences closely resemble those recorded in a range of wrasse species on coral reefs (Fulton & Bellwood, 2002). Additionally, cases of food distribution affecting foraging strategies, like those seen here, have been seen in a variety of systems, including ant colonies (Lanan, 2014). However, we currently lack detailed information on the spatial organization of algal resources needed to determine the exact relationships between resource distributions and the fishes' foraging movements. Differences in foraging paths may be influenced by many aspects of the targeted food resources, including their patchiness, within patch density, or their nutritional and energetic quality (Schatz & McCauley, 2007).

While both red algae and cyanobacteria are thought to be nutritionally poor, cyanobacteria appear physically less dense than corticated red algae, lacking the same hard external tissues. A lack of hard tissues could make cyanobacteria easier to mechanically process when feeding, consistent with observations of larger handling times for crustacean prey (Hoyle & Keast, 1987). Under the patch model of optimal foraging theory, a foraging strategy involving long travel to distant patches is linked with low quality of nearby patches (Charnov, 1976). A forager will leave a patch and continue searching when the rate of energy gain in a patch has been reduced below what could be obtained elsewhere (Stephens & Krebs, 1986). If cyanobacteria are particularly easy to process, then the “quality” (here related directly to quantity) of cyanobacteria patches may be reduced sooner than that of red algae, prompting patch exit and further exploration. These differences could result in a foraging strategy with shorter patch residence times, and larger territory sizes on average (Charnov, 1976; Stephens & Krebs, 1986). Corticated algae patches on the other hand may maintain their quality long enough to favor long patch occupancy, and smaller range sizes.

Another difference between these two food sources is that cyanobacteria are considered unpalatable for many species and produce metabolites to deter their consumption (Capper et al., 2006; Paul et al., 1990, 1992). Toxin constraint models predict foragers should exhibit partial food preferences, consuming multiple food types even when a toxin‐producing food item is most nutritionally profitable (Stephens & Krebs, 1986). In this way profitability of food items will be balanced against toxins they contain. This balance was illustrated for reef herbivores in a study by Hay et al. (1994) where, when given a choice between a control food source and one supplemented with metabolites, reef and seagrass parrotfishes almost exclusively consumed the control food sources. Because of this, *Si. vulpinus* may need to supplement its diet with other food sources that, while less preferred, produce less toxin. For instance, dense, mat‐forming species of cyanobacteria (e.g., genus *Lyngbya*) are expected to produce more toxins than their sparser counterparts (Cissell et al., 2019). Consequently, short patch residence times and wide movements for *Si. vulpinus* may be due to the quicker depletion of less‐dense cyanobacteria patches that produce less toxin. However, without similar choice experiments on these species, it is unclear how much rabbitfishes are constrained by cyanobacterial metabolites.

The feeding efficiency approach given here reveals some intriguing differences between species. However, without clear info on nutritional content and assimilation efficiency these comparisons are solely exploratory. Energy budgets are complex and, in addition to these nutritional factors, are a result of other properties like body size and swimming style/speed. The two families differ substantially in their locomotion: while rabbitfishes rely largely on undulating caudal and pectoral‐caudal propulsion, wrasses (such as parrotfishes) almost exclusively use flapping pectoral propulsion (Fulton, 2007). Energetic studies have suggested that flapping, pectoral propulsion (labriform swimming) is more energy efficient than undulating (Korsmeyer et al., 2002) or rowing pectoral propulsion, the latter of which rabbitfishes frequently employ for fine‐scale maneuvering (Jones et al., 2007). Thus, in principle, one may expect that the parrotfish could meet energetic demands with lower feeding efficiency than the two rabbitfish species. Nevertheless, there are important other considerations that can underpin energetic demands, such as energy and nutrient content of food items. First, given the strong relationship between body mass and metabolism, a *Sc*. *schlegeli* of 20 cm (192 g, estimated using length‐weight relationships) would have a resting metabolism approximately nearly 50% higher than than a *Si. corallinus* of equal length (117 g) and would require substantially more energy (Clarke & Johnston, 1999). Second, by scraping microbes from the calcareous reef matrix and winnowing through unwanted material, energetic and nutritional net gains per bite may be low for the parrotfish (Clements et al., 2016), thus necessitating high ration of bites per unit distance covered during foraging despite the lower energetic demands of labriform locomotion. In contrast, procurement of algae may be relatively easy for the two rabbitfishes. Our findings highlight the important need to investigate reef herbivores through an energetic and nutritional lens to fully understand the drivers and consequences of their foraging patterns.

Protecting valuable ecosystem functions requires an understanding of variations within and between functional entities (Brandl et al., 2019). Our work highlights the importance of foraging behavior as an important dimension in species management, as nuanced behavioral differences among fish species can indicate strong species‐specific patterns of space and resource use that can result in complementarity in functional roles. This complementarity is ultimately driven by differences in species' energy budgets, which emphasizes the need for detailed examinations of consumer species, their food choices, and the functional consequences of this interaction.

## CONFLICT OF INTEREST

The authors declare no conflicts of interest exist.

## AUTHOR CONTRIBUTION

**Robert F. Semmler:** Conceptualization (equal); Formal analysis (equal); Visualization (lead); Writing‐original draft (lead); Writing‐review & editing (equal). **Simon J. Brandl:** Conceptualization (equal); Data curation (lead); Formal analysis (equal); Methodology (equal); Supervision (equal); Writing‐original draft (supporting); Writing‐review & editing (equal). **Sally A. Keith:** Supervision (equal); Writing‐review & editing (supporting). **David R. Bellwood:** Funding acquisition (lead); Methodology (supporting); Resources (lead); Supervision (supporting); Writing‐review & editing (equal).

## ETHICAL APPROVAL

All work was conducted in with accordance with JCU ethics standards on animal research, research was approved under JCU Ethics Approvals A1700 and A2086.

## Data Availability

The feeding path and active tracking datasets analyzed in this study are available in the FigShare repository (https://doi.org/10.6084/m9.figshare.12233579) and (https://doi.org/10.6084/m9.figshare.12233567).
